# Critical aspects of substrate nanopatterning for the ordered growth of GaN nanocolumns

**DOI:** 10.1186/1556-276X-6-632

**Published:** 2011-12-14

**Authors:** Francesca Barbagini, Ana Bengoechea-Encabo, Steven Albert, Javier Martinez, Miguel Angel Sanchez García, Achim Trampert, Enrique Calleja

**Affiliations:** 1 ISOM and Electrical Engineering Dept. (DIE), Escuela Técnica Superior de Ingenieros de Telecomunicaciónes (ETSIT), Universidad Politécnica de Madrid s/n, Madrid, 28040, Spain; 2 Paul Drude Institut für Festköperelektronik, Hausvogteiplatz 5-7, Berlin, 10117, Germany

**Keywords:** GaN nanocolumns, ordered growth, molecular beam epitaxy, surface cleaning, roughness, adhesion, e-beam lithography, colloidal lithography, focused ion beam

## Abstract

Precise and reproducible surface nanopatterning is the key for a successful ordered growth of GaN nanocolumns. In this work, we point out the main technological issues related to the patterning process, mainly surface roughness and cleaning, and mask adhesion to the substrate. We found that each of these factors, process-related, has a dramatic impact on the subsequent selective growth of the columns inside the patterned holes. We compare the performance of e-beam lithography, colloidal lithography, and focused ion beam in the fabrication of hole-patterned masks for ordered columnar growth. These results are applicable to the ordered growth of nanocolumns of different materials.

## Background

The unique properties of III-nitride nanocolumns [NCs] in contrast to thin film structures derive from the reduced footprint on the substrate that enables essentially dislocation- and strain- free growth on a variety of substrates [1]. Defect-free NCs exhibit excellent electronic transport and optical properties for the fabrication of high-efficiency optoelectronic nanodevices, such as photodetectors, light-emitting diodes, and solar cells [2-5]. Moreover, the controlled coalescence of III-nitride NCs would lead to strain-free pseudosubstrates with reduced defect densities [6].

During the past years, III-nitride NCs have been grown in the self-assembled mode by plasma-assisted molecular beam epitaxy [PA-MBE] [7-9] on various substrates. However, fluctuations in density and dimensions of the NCs lead to significant dispersion in the optoelectronic properties and render the device processing very difficult. Thus, the realization of true devices relies on the achievement of ordered arrays of homogeneous NCs by localization of the epitaxial growth on predetermined preferential sites. This growth mode is known as *selective area growth *[SAG], and it has attracted much scientific interest in the last few years [10-15].

In the SAG, the substrate is pre-patterned with a mask of nanoholes. The NCs nucleate and grow selectively inside the nanoholes and not on the surface of the mask. Many experimental works have been reported on the SAG of GaN/InGaN nanocolumnar heterostructures [10-14]. In these works, the hole patterning of the mask material was achieved either by focused ion beam [FIB] or by e-beam lithography [EBL]. However, despite the fundamental importance of the nanopatterning process, no detailed information has been reported neither on the choice of the particular nanopatterning technique nor on the importance of the morphology of the patterned mask with respect to the subsequent selective growth. Within this context, the quality of the surface pattern is crucial because it determines whether selectivity is achieved or not.

This work studies the hole patterning of the mask material for the subsequent SAG of GaN NCs by PA-MBE. Three different techniques are reported that were successfully used to pre-pattern the surface of a thin Ti mask with ordered arrays of nanoholes: EBL followed by dry etch, colloidal lithography [CL], and FIB. The critical issues encountered in the mask fabrication processes are studied in detail. More specifically, the effects of surface roughness, adhesion of the mask layer to the substrate, and surface cleanliness on the following GaN NCs SAG are analyzed. For each of the mentioned techniques, the main advantages and drawbacks are highlighted. Only when the patterning process was optimized, high-quality hole-patterned masks of different dimensions and geometry were obtained. These masks were subsequently used to grow ordered crystalline GaN NCs in the SAG mode by PA-MBE. The technological issues discussed in this work can be applied to the ordered growth of any kind of material on various substrates.

## Methods

### Substrate nanopatterning

All substrates used in this work were commercial 2-in. wafers consisting of a 4-μm GaN (0001) layer grown on sapphire by MOVPE (Lumilog, Les Moulins, Vallauris, France). These substrates were cleaned in *N*-methyl-pyrrolidone [NMP] at 90°C for 30 min, rinsed in isopropanol [IPA], and thoroughly cleared with deionized [DI] water. The mask material always consisted of a 5- to 10-nm Ti layer deposited on a clean GaN template by e-beam evaporation. The root mean square [RMS] roughness of the wafer surface before and after Ti deposition was 0.4 ± 0.1 nm in an area of 1 μm^2^. The various techniques used to pattern the Ti mask with ordered arrays of nanoholes are described in detail in the following subparagraphs. Prior to each PA-MBE growth, the distribution, diameter, and depth of the nanoholes were characterized by atomic force miscroscopy [AFM] (Nanoscope III Multimode AFM, Veeco Instruments Inc., Plainview, NY, USA) and scanning electron microscopy [SEM] (CABL-9500C, Crestec Co. Ltd., Hamamatsu, Shizuoka, Japan).

#### E-beam lithography

A 350-nm layer of positive resist ZEP520A (Zeon Co., Tokyo, Japan) was spun at 4,000 rpm on the Ti mask. After baking at 190°C for 2 min, the sample was transferred to the EBL system. The nanoholes were opened in the resist using a current of 0.5 × 10^-9 ^A, and an exposure time ranging from 15 to 45 μs. The process conditions were optimized to obtain arrays of nanoholes with diameters ranging from 50 to 200 nm and pitch (center-to-center distance) varying from 80 nm to 300 nm. After developing the resist, the sample was dry-etched in O_2_/CF_4 _plasma at 10^-2 ^mbar and 110 W (Plasmalab, Oxford Instruments plc, Abingdon, Oxfordshire, UK) for 130 s. The post-etch residue was removed by immersing in NMP at 80°C for 30 min and abundantly rinsing in IPA and DI water. In some cases, oxygen plasma was necessary to completely remove the resist hardened by the previous plasma etching. AFM analysis revealed an etching depth between 7 and 10 nm; thus, the GaN material underneath the Ti mask was also etched some 2 to 5 nm.

#### Colloidal lithography

This method was extensively introduced elsewhere [16]. Briefly, the GaN substrate was made negatively charged by coating with a trilayer of polyelectrolytes. Monodispersed sulfate latex spheres (mean diameter of 260 nm; Invitrogen, Carlsbad, CA, USA) were spun on GaN from aqueous solutions to obtain a densely packed monolayer of nanospheres. Subsequent oxygen plasma was used to reduce the sphere dimensions thus creating some sphere-to-sphere interspace. A 5- to 10-nm Ti layer was evaporated on top, and the spheres were finally stripped from the sample using an adhesive pad. The final cleaning step consisted of using NMP at 90°C to dissolve any latex residue from the nanoparticles and thoroughly rinsing with DI water.

#### Focused ion beam

The nanoholes were opened in the 7-nm Ti mask on GaN by a focused ion beam in a one-step process. The liquid-metal ion source used was Ga^+ ^at 30 KeV (ionLine Raith GmbH, Dortmund, Germany). The process conditions were optimized to obtain arrays of nanoholes with a 100-nm diameter and 250-nm pitch (30 pA, ion dose 10^17 ^cm^-2^). No extra cleaning steps were applied after the ion etching. AFM analysis revealed an etching depth between 10 and 15 nm. Little redeposition of Ti (1 to 2 nm) appears in some cases at the edges of the holes.

### Ordered nanocolumnar growth

GaN NCs were grown on the hole-patterned masks using radio frequency [RF] PA-MBE (Compact 21, Riber, USA). The substrate temperature during growth was measured with a thermocouple located at the growth stage. The Ga and N fluxes were calibrated in equivalent (0001) GaN growth rate units for compact layers in nanometers per minute, which are the standard units used in nitride PA-MBE growth diagrams. The Ti mask was nitrided prior to growth to prevent its degradation due to the high temperatures used in GaN NCs SAG (860°C to 900°C). We used a two-step nitridation process, as proposed by Sekiguchi et al. [10,11]: 10 min at 460°C followed by 3 min at 880°C. During nitridation, the plasma power was set to 580 W and the nitrogen flux, to 1.2 sccm. These conditions correspond to an equivalent stoichiometric GaN growth rate higher than 30 nm/min. Electron energy loss spectroscopy measurements proved the formation of TiN, which is more stable than Ti at high temperatures. During the growth phase, we lowered the plasma power and the nitrogen flux to 150 W and 0.3 sccm, respectively. The GaN flux is maintained at a corresponding GaN growth rate of 16 nm/min. These conditions resulted in a highly selective ordered growth inside the nanoholes, as widely illustrated in a previous publication [13].

## Results and discussion

The mask fabrication process consists of many critical steps that, in the worst case scenario, might lead to the total failure of the selective growth. The factors to account for can be basically summarized into surface roughness, surface cleaning, and adhesion of the mask material to the substrate. Each of them is treated separately in the following subparagraphs.

### Surface roughness

**Figure 1:**
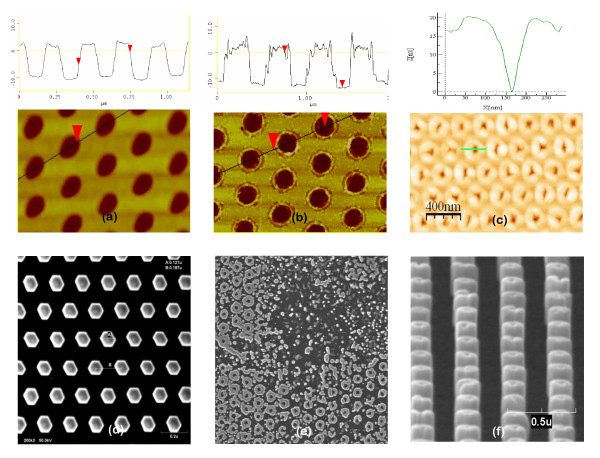
**Influence of surface roughness on the selective columnar growth**. Top row: typical example of (**a**) an ideal smooth mask obtained by EBL, (**b**) masks with increased roughness in the area around the holes obtained by EBL, and (**c**) a FIB mask with bumps around the holes. Insets: cross-section AFM analysis of the nanoholes. Bottom row: growth results using the respective masks in the upper row. (**d**) With an ideal smooth mask, perfect ordered growth is achieved; in the case of local higher roughness around the holes, typical donut-shaped growth is observed from the (**e**) top view and (**f**) side view SEM images.

Figure 1 shows the case of a smooth EBL mask surface (Figure 1a), the case of an EBL mask with local increase in roughness in the 20-nm region around the holes' rim (Figure 1b), and the case of a FIB mask with bumps in the 20-nm region around the holes' rim (Figure 1c). In the case of Figure 1a, the surface roughness of the mask as measured by AFM is 0.5 ± 0.1 nm, which is similar to the RMS values of both the bare GaN template and the as-deposited Ti layer. These roughness values lead to PA-MBE growth with a perfect (100%) selective nucleation of NCs inside the holes (Figure 1d). On the contrary, accurate AFM analysis of the masks in Figure 1b, c revealed the presence of material around the holes' rim that locally increased the surface roughness by 1 nm or higher. In the case of EBL masks (Figure 1b), this particular morphology is attributed to the redeposition of etched material around the holes during the plasma etch, whereas surface bumps around the holes in FIB masks are probably formed when bombarding the Ti with a high Ga dose. Both these cases resulted in a typical donut-shaped growth, where many thin NCs nucleate around the holes' rim leaving the holes core empty, as shown in Figure 1e, f. In about 50% of the cases, single tubular NCs were observed, as shown in Figure 1f. It was demonstrated [17] that surface migration of ad-atoms is the main contribution for selective growth when using Ti masks. This peculiar growth layout is thus likely due to the difficulty for the Ga and N ad-atoms to diffuse from the surface into the holes due to either the deposited material or bumps that act as diffusion barriers. For this reason, the NCs nucleate at the perimeter of the donut-shaped bumps. The presence of accumulated material around the holes can only be detected by accurate AFM analysis, while SEM pictures show smooth surfaces even when there is local increase in RMS. To minimize this material redeposition and achieve optimal roughness conditions, we optimized both the plasma etch in EBL technique (mainly plasma power and time) and the dose in the FIB. In particular, the EBL and etch processes were optimized using three series of samples. First, the EBL dose was varied until finding the minimum dose required to open sufficiently wide holes that subsequently enabled to completely etch the Ti layer underneath. In the second series of samples, we thus used this optimal EBL conditions and increased the RF power during etch, until the material redeposition around the holes was minimized. In the last series of samples, we maintained constant EBL conditions and RF power during the etching while slightly reducing the etching time in order to leave the surface roughness as low as possible and ensuring to etch the complete Ti layer and 1-2 nm of the underneath GaN material. SEM and AFM studies of the holes geometry and of the surface roughness were measured at the end of the complete processing for each series of samples. For the FIB optimization, several dose studies were necessary in order to obtain the best conditions to pattern holes with a depth of 7 nm. An array of holes with similar diameter (100 nm) but different doses was patterned on a small piece of Ti (7-nm thickness) on GaN. After the patterning, the depth of the nanoholes and the surface roughness of the Ti were measured by AFM. The hole with the right depth and no bumps at the surface was considered as the optimal dose for the FIB. In our case, the optimal ion dose was 10^17 ^cm^-2^. The surface roughness was never an issue when preparing the masks by CL since there was no etching involved.

### Surface cleaning of the hole-patterned mask

**Figure 2:**
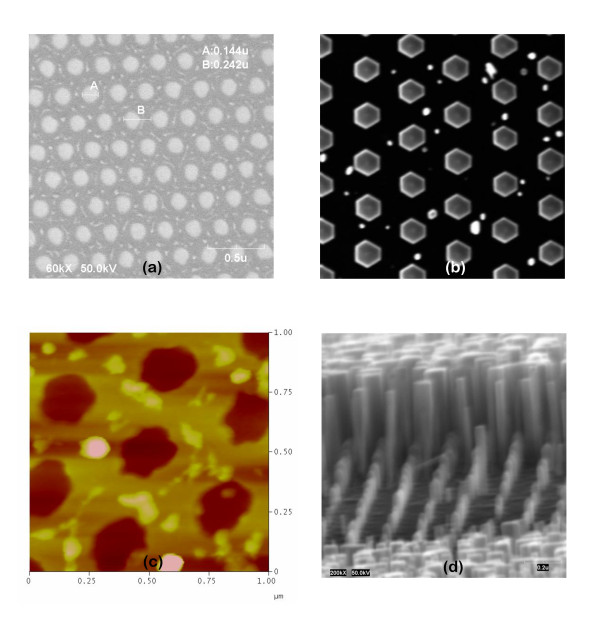
**Effect of surface cleaning on the columnar growth**. Left side: (**a**) SEM and (**c**) AFM images of a nanopatterned Ti mask with resist residues (whiter spots). Right side: (**b**) top view and (**d**) side view SEM images of GaN NCs grown by PA-MBE using these masks, showing ordered columns grown inside the holes and thinner and longer columns grown in the area between the holes on the Ti mask.

When the surface of the patterned mask present resist residues, as shown in Figure 2a, c (SEM and AFM, respectively), GaN NCs still nucleate inside the holes and exhibit constant distribution, height, and diameter. However, in the spots where the resist residues lie, thinner NCs nucleate on the mask and grow faster than the ordered ones inside the holes. These growth results are displayed in Figure 2b, d (top view and side view, respectively). In principle, organic contaminants, including resist residues, should evaporate during the nitridation at high temperature. However, similar growth features were always observed when some resist residues were present on the mask before the growth. In addition to this, we highlight in general on the detrimental effect of organic contamination on the subsequent growth. This point sounds trivial, though it is not always straightforward to identify resist residues over the mask or inside the holes, due to the high aspect ratio of the patterns and to the finite dimensions of the AFM tip. In 50% of the cases, neither SEM nor AFM analysis of the patterned mask showed any surface contamination before the growth. However, from the growth results, we deduced that contamination occurred at some point during the mask fabrication. When organic contamination occurred inside the holes only, non-uniform growth was observed, where the contaminated holes exhibited no nucleation at all.

Surface cleaning is thus a key point to consider in any method of mask preparation. However, the plasma-hardened photoresist is very difficult to remove compared to common organic contaminants. For this reason, the issue of surface cleaning becomes even more important when preparing the masks by EBL. The optimal cleaning process for EBL masks before loading the sample in the PA-MBE chamber consisted of 30 min immersion in pyrrolidone at 80°C followed by a hot isopropanol rinse and abundant hot water rinse. Finally, a 15-s oxygen plasma was performed.

### Adhesion of the Ti mask to the substrate

**Figure 3:**
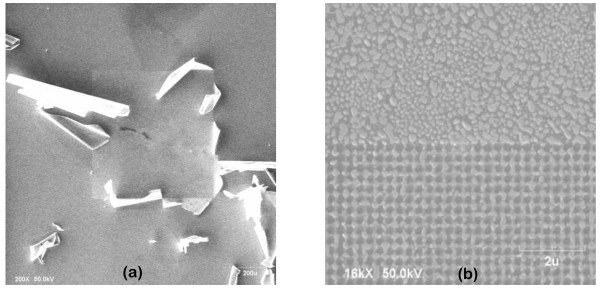
**Effect of titanium mask quality on the columnar growth**. (**a**) Peeling-off of a 10-nm thick Ti mask after PA-MBE growth. (**b**) Degradation of a 5-nm thick Ti mask during nitridation at high temperature in the PA-MBE chamber.

A thick Ti layer barely adhered to the GaN substrate material resulted in delamination of the mask during the MBE growth, as shown in Figure 3a. This delamination was observed when the PA-MBE growth stage cooled down after growth from 650°C to 700°C, to room temperature. This issue was solved by optimizing both the adhesion of the Ti film to the substrate and the thickness of the Ti film. To improve adhesion, the initial GaN template (as received) was cleaned with NMP at 90° by sweeping the surface several times with a soft stick, then abundantly rinsing with IPA and DIW. Prior to Ti deposition, the substrate was heated up in an oven at 300°C for 30 min to desorb water molecules from the surface. The optimal thickness of the Ti layer was found empirically. Since the mask adhesion was optimized, delamination was probably due to the strain generated by thermal shock when cooling down the system. To decrease the amount of strain, the thickness of the Ti mask was reduced from 10 nm to 7 nm. A thinner mask resulted in most cases in the complete degradation of the Ti material at high temperature during the growth, as shown in Figure 3b. Mask adhesion to the substrate is a factor to consider in all of the proposed methods, meaning EBL, FIB, and CL. Both optimal substrate cleaning and mask thickness are of fundamental importance for a good adhesion of the Ti layer to the substrate.

**Figure 4:**
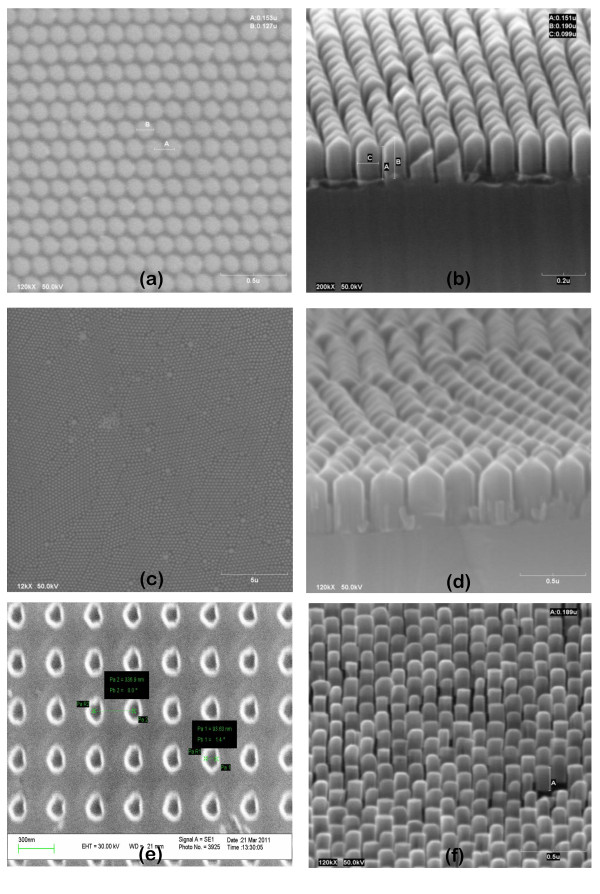
**High quality nanopatterned masks resulting in ordered columnar growth**. Left side: ordered arrays of nanoholes on Ti masks on GaN template, obtained by (**a**) EBL, (**c**) CL, and (**e**) FIB. Right side: typical results (**b**, **d**, and **f**) of the selective area growth of GaN NCs by PA-MBE on each of the respective masks.

Despite all these technical issues, high-quality nanopatterned masks were successfully fabricated using all of the three techniques by optimizing the process parameters. Typical examples of hole-patterned Ti masks as obtained by EBL, CL, and FIB are shown in Figure 4a, c, e, respectively. In all cases, the diameter of the holes varies between 50 nm and 250 nm, and the pitch, between 80 nm and 350 nm. Using these masks, selective nucleation and growth of GaN NCs inside the holes were achieved by PA-MBE, independent of the characteristic mask dimensions (within the above mentioned range) and of the particular patterning technique. These growth results are shown in Figure 4b, d, f for each of the respective masks. Ordered GaN NCs always exhibited a constant diameter and interdistance that fit to the geometry of the initial patterned mask, meaning that the vertical growth rate was much higher than the lateral one. After 30 min of growth, all the GaN NCs have a constant height of about 200 nm and show a perfect hexagonal cross section. The latter can be better appreciated in Figure 1d, which shows a top view of the ordered NCs. High ordering and constant geometrical parameters (pitch, diameter) were easily achieved in the case of both EBL and FIB. In the case of colloidal lithography, however, we stress on the extraordinary level of ordering and homogeneity in the diameter of the holes and pitch over large areas of several microns, despite the simplicity of the process. Only localized areas show little defects such as a larger hole or a missed hole. These defects are due to agglomeration of particles during deposition or to a missed particle in the initial nanosphere monolayer.

**Figure 5:**
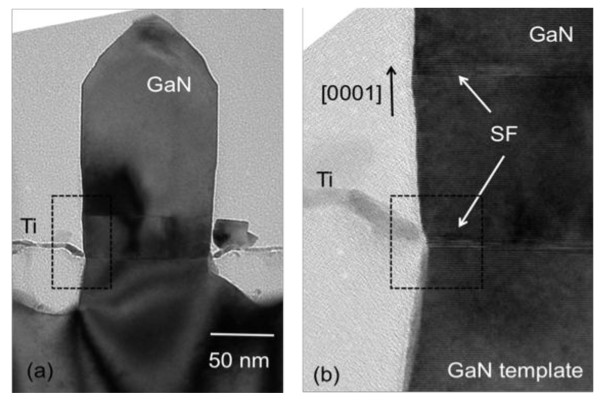
**TEM analysis of a single GaN nanocolumn**. (**a**) Cross-sectional bright-field TEM image, showing a single ordered GaN NC. (**b**) Higher magnification of the column/GaN/Ti interface, where stacking faults are visible from the diffraction contrast (dark lines).

Figure 5a, b shows the TEM analysis of a single GaN NC. In particular, Figure 5b is a magnification at the NC/GaN template/Ti interface. The diffraction contrast image, which is sensitive to crystal defects, exhibits dark contrast lines that give evidence for the presence of stacking faults at the NC/GaN template interface. However, it is clearly shown that the stacking faults cross the whole rod and do not lead to the formation of partial dislocations. The small footprint and the large surface area (high aspect ratio) of NCs are known to induce strain-free growth. Initial dislocations, either from the substrate or generated during the nucleation stage, bend to the lateral surface [1,2]. Moreover, the initial GaN template consisted of a 4-μm strain-free GaN layer on sapphire, and the NCs growth is homoepitaxial. For all the mentioned reasons, we conclude that there is no epitaxial strain involved. The presence of stacking faults at the bottom of the NCs could be attributed to impurities, such as Ti.

From these results, it becomes evident that high-precision ordering of GaN NCs can easily be achieved by either EBL or FIB techniques. The main disadvantage of the EBL technique is the need for etching and the organic-cleaning steps. Both these steps, when not optimized, increase the surface roughness and/or the level of surface contamination, leading to a total failure of the selective growth. In contrast, FIB and CL techniques require neither the etch step nor a strong post-processing surface cleaning. However, the FIB process must be optimized to prevent the formation of bumps around the holes that block the surface diffusion of ad-atoms and hence the SAG. Finally, CL enables to obtain large patterned areas using a low-cost, fast processing. The main drawback of this technique is the lack of a precise predefined ordered patterning.

## Conclusions

Optimized EBL, FIB, and CL processes were used to fabricate high-quality masks patterned with nanoholes, which served as nucleation sites for the selective area growth of GaN NCs. Once the process window for the ordered growth of GaN NCs by PA-MBE was identified, the successful selective growth was driven by the morphology of the hole-patterned Ti mask. Surface roughness and cleaning, and adhesion of the Ti mask to the GaN substrate are the most critical aspects that might negatively influence the ordered growth. In this context, our results suggest that FIB and CL, where neither etching steps nor organic chemicals are introduced, are preferred techniques to fabricate high-quality and reproducible hole-patterned masks. CL in particular is easy and fast compared to any other method. However, it lacks a predefined high-precision mask layout. FIB might present the issue of material redeposition around the patterns. Although this paper focuses on GaN nanocolumns, these technological aspects can be extended to the selective growth of nanostructures of different material and geometries.

## Abbreviations

AFM: atomic force microscopy; CL: colloidal lithography; DI: deionized; EBL: electronic beam lithography; FIB: focused ion beam; IPA: isopropanol: NCs: nanocolumns; NMP: normal-methyl-pyrrolidone; PA-MBE: plasma-assisted molecular beam epitaxy; RF: radio frequency; SAG: selective area growth: SEM: scanning electron microscopy; TEM: transmission electron microscopy.

## Competing interests

The authors declare that they have no competing interests.

## Authors' contributions

FB performed the substrate nanopatterning by e-beam lithography. ABE and SA carried out the PA-MBE growths. JM provided the mask patterned by FIB. MASG supervised the PA-MBE growth. EC supervised the whole work as the principal scientist. AT performed the TEM analysis. All authors read and approved the final manuscript.
